# Exogenous melatonin improves the salt tolerance of cotton by removing active oxygen and protecting photosynthetic organs

**DOI:** 10.1186/s12870-021-03082-7

**Published:** 2021-07-10

**Authors:** Dan Jiang, Bin Lu, Liantao Liu, Wenjing Duan, Yanjun Meng, Jin Li, Ke Zhang, Hongchun Sun, Yongjiang Zhang, Hezhong Dong, Zhiying Bai, Cundong Li

**Affiliations:** 1 State Key Laboratory of North China Crop Improvement and Regulation/College of Life Science, Hebei Agricultural University, Baoding, 071001 Hebei China; 2 State Key Laboratory of North China Crop Improvement and Regulation/Key Laboratory of Crop Growth regulation of Hebei Province/College of Agronomy, Hebei Agricultural University, Baoding, 071001 Hebei China; 3 College of Landscape and Tourism, Hebei Agricultural University, Baoding, 071001 China; 4 Cotton Research Center/Key Laboratory of Cotton Breeding and Cultivation in Huang-huai-hai Plain, Ministry of Agriculture, Shandong Academy of Agricultural Sciences, Jinan, 250100 Shandong China

**Keywords:** Melatonin, Salt stress, Photosynthesis, Stomata, ROS, Chloroplast, Anatomical structure

## Abstract

**Background:**

As damage to the ecological environment continues to increase amid unreasonable amounts of irrigation, soil salinization has become a major challenge to agricultural development. Melatonin (MT) is a pleiotropic signal molecule and indole hormone, which alleviates the damage of abiotic stress to plants. MT has been confirmed to eliminate reactive oxygen species (ROS) by improving the antioxidant system and reducing oxidative damage under adversity. However, the mechanism by which exogenous MT mediates salt tolerance by regulating the photosynthetic capacity and ion balance of cotton seedlings still remains unknown. In this study, the regulatory effects of MT on the photosynthetic system, osmotic modulators, chloroplast, and anatomical structure of cotton seedlings were determined under 0–500 μM MT treatments with salt stress induced by treatment with 150 mM NaCl.

**Results:**

Salt stress reduces the chlorophyll content, net photosynthetic rate, stomatal conductance, intercellular CO_2_ concentration, transpiration rate, PSII photochemical efficiency, PSII actual photochemical quantum yield, the apparent electron transfer efficiency, stomata opening, and biomass. In addition, it increases non-photochemical quenching. All of these responses were effectively alleviated by exogenous treatment with MT. Exogenous MT reduces oxidative damage and lipid peroxidation by reducing salt-induced ROS and protects the plasma membrane from oxidative toxicity. MT also reduces the osmotic pressure by reducing the salt-induced accumulation of Na^+^ and increasing the contents of K^+^ and proline. Exogenous MT can facilitate stomatal opening and protect the integrity of cotton chloroplast grana lamella structure and mitochondria under salt stress, protect the photosynthetic system of plants, and improve their biomass. An anatomical analysis of leaves and stems showed that MT can improve xylem and phloem and other properties and aides in the transportation of water, inorganic salts, and organic substances. Therefore, the application of MT attenuates salt-induced stress damage to plants. Treatment with exogenous MT positively increased the salt tolerance of cotton seedlings by improving their photosynthetic capacity, stomatal characteristics, ion balance, osmotic substance biosynthetic pathways, and chloroplast and anatomical structures (xylem vessels and phloem vessels).

**Conclusions:**

Our study attributes help to protect the structural stability of photosynthetic organs and increase the amount of material accumulation, thereby reducing salt-induced secondary stress. The mechanisms of MT-induced plant tolerance to salt stress provide a theoretical basis for the use of MT to alleviate salt stress caused by unreasonable irrigation, fertilization, and climate change.

## Background

Photosynthesis is an indispensable physiological activity for plant growth and development. It is closely related to chlorophyll content, chloroplast structure, and osmotic adjustment substances and is extremely susceptible to the influence of external environment [1]. Salt stress is one of the primary adversities that affect plant photosynthesis. The area of saline-alkali land in the world is increasing every year with the continuous changes in the ecological environment, and soil salinization has become a major obstacle to agricultural development [2]. Currently, the area of saline-alkali land in China is approximately 1.0 × 10^8^ hm^2^, and its trend is toward overall deterioration and an increase in area. It has become one of the important factors that affect the sustainable development of agricultural production and ecological environment in China [3]. When plants are in a medium-high salt environment, they will suffer three kinds of stress, including ion imbalance, osmotic stress and oxidative damage.

The first stage of effect of salt stress on cotton is osmotic stress [2], in which the ability of plant to absorb water is reduced, leading to cell dehydration and partial closure of the stomata, reducing cell swelling in the stem and young leaves [4, 5]. Under salt stress conditions, salt ions accumulate excessively in old leaves through transpiration, causing changes in cytotoxicity and ion homeostasis and leading to ionic stress [6]. In addition, osmotic stress and ionic stress can affect plant morphology and physiological and biochemical processes, thus, leading to the inhibition of plant growth and even death [7]. Salt stress directly affects the stomata and gene expression, leading to stomatal closure and reducing the intake of carbon dioxide, which in turn, reduces the net photosynthetic rate [7–9]. Plants can accumulate excessive reactive oxygen species (ROS), including hydrogen peroxide (H_2_O_2_) and superoxide anions (${\mathrm{O}}_2^{\cdotp -}$), under the combined action of osmotic and ionic stress. Excessive ROS can cause cell damage and oxidative stress to plants [5], and damage to DNA, enzymes and biofilms, thus, affecting plant growth and development [10]. Photosynthesis is an important physical and chemical process responsible for the production of higher energy plants, which may be influenced by ROS, leading to a loss in plant production [11]. Chloroplasts in mesophyll cells are the organelles most sensitive to salt stress [12]. With the increase of salt concentration, the structure of chloroplast begins to be damaged to varying degrees [13]. Relevant studies have found that salt stress leads to disordered and enlarged thylakoid arrangement, blurred boundaries between grana and stroma lamella, damage or disappearance of the membrane, and even disintegration [14–16].

Cotton is a field crop for both cotton and firewood and occupies an important position in the national economy of China and that of the world [17]. The seedling stage is a fragile and critical period in the growth and development of cotton that is susceptible to biotic and abiotic stress, and it is greatly affected by salt stress. Therefore, it is highly important to explore the effects of salt stress on photosynthesis and related factors of cotton seedlings.

Melatonin (N-acetyl-5-methoxytryptophan, MT), also known as pinealin, is a hydrophilic and lipophilic physiological regulator, which plays an important role in the response to scavenging free radicals, the maintenance of membrane integrity, and the prevention of chlorophyll degradation [18]. MT was first discovered in vascular plants in 1995, among which Dubbels et al. [19] used gas chromatography-mass spectrometry technology to confirm that the leaf tissues of tomatoes, bananas, cucumbers and tobacco all contain melatonin. Hattori et al. [20] used radioimmunoassay technology to determine the melatonin content of 24 edible plants in 12 families of dicotyledons and monocotyledons. The plants tested all contained melatonin. Subsequent research found that mitochondria and chloroplasts, semi-autonomous organelles in plants, are the sites of synthesis of MT [21]. Studies have shown that MT is widely present in almost all plant species and in various organs of higher plants [22, 23], and the concentration varies with the plant tissue, developmental period, light and environmental differences [24]. Subsequent studies have shown that MT plays a role in the regulation of plant growth and developmental stages [25–29], and the alleviation of abiotic stress [30–34].

Melatonin can directly neutralize ROS, and also stimulate the antioxidant system to increase its efficiency; therefore, it is called an endogenous free radical scavenger [35, 36]. Studies have found that melatonin regulates diverse biological responses of plants and improves plant tolerance to adversity. Melatonin protects the photosynthetic capacity [37], reduces the osmotic stress and ion toxicity [38–41], improves the characteristics of stomata [38], protects chloroplast structure, grana lamellae, more complete mesophyll cells and more chloroplasts [42, 43], and improves the anatomical structure of plants [44, 45] under stress. Studies on maize (*Zea mays*) [46], rapeseed [47], soybean [48] and tea [49] have shown that MT alleviates the damage to plants caused by stress. The application of MT has been shown to promote an increase in the activities of cotton antioxidant enzymes, and increase the plant height and leaf area of cotton seedlings [50], which may be owing to the appropriate concentration of MT. It increases the chlorophyll content and photosynthetic capacity of leaves under salt stress, alleviates the oxidative inhibition caused by salt stress, and promotes plant growth. However, the manner by which exogenous MT alleviates the damage of salt stress on the photosynthesis of cotton seedlings remains unclear. In this experiment, cotton seedlings were grown in a hydroponic solution without salt (150 mM) or with salt (150 mM) and sprayed with MT. The purposes of this study were to (1) clarify the mechanism of MT on the photosynthetic and fluorescence characteristics, (2) elucidate the regulatory effect of MT on reactive oxygen species, proline, and ions, and (3) explore the regulatory effect of MT on the structure of stems, leaves, and chloroplasts in cotton seedlings. The results of this experiment provide theoretical support to promote the application of exogenous melatonin in saline-alkali cotton, increasing crop growth and improving product quality.

## Results

### Melatonin improved the biomass and chlorophyll content in cotton seedling under salt stress

The fresh weight (FW) and dry weight (DW) of shoots and roots of the seedlings were significantly reduced under the salt (S) treatment (Table 1). Compared with the CK treatment, the FW of shoots and roots was reduced by approximately 37% and 36%, respectively, under salt treatment, and the DW of shoots and roots was reduced by approximately 40% and 35%, respectively. In contrast, treatment with exogenous MT reduced the inhibitory effect of salt stress on the growth of cotton seedlings. The FW of the shoots and roots of cotton seedlings treated with 200 μM MT increased by 36% and 48%, respectively. The DW increased by approximately 40% and 49%, respectively. Compared with the CK treatment, under salt treatment, the DW of total (TDW) plant was reduced by 39%; the TDW of cotton seedlings treated with 200 μM MT increased by 41%, and the treatment resulted in more pronounced growth of cotton seedlings.

**Table 1:** Effects of melatonin on the morphology of cotton seedlings under salt stress for 12 days

Treatment	Shoot fresh weight (g plant^−1^)	Shoot dry weight (g plant^−1^)	Root fresh weight (g plant^−1^)	Root dry weight (g plant^−1^)	The total dry weight (g plant^−1^)
CK	10.52 ± 0.43 a	1.16 ± 0.05 a	4.23 ± 0.48 a	0.26 ± 0.01 a	1.43 ± 0.06 a
S	6.62 ± 0.38 d	0.69 ± 0.06 e	2.69 ± 0.32 d	0.17 ± 0.01 d	0.86 ± 0.07 e
S + MT50 S + MT100 S + MT200 S + MT500	7.06 ± 0.22 d 7.82 ± 0.19 c 9.01 ± 0.58 b 7.68 ± 0.18 c	0.75 ± 0.01 de 0.84 ± 0.05 c 0.97 ± 0.05 b 0.81 ± 0.05 cd	2.87 ± 0.14 d 3.45 ± 0.37 c 3.99 ± 0.11 ab 3.48 ± 0.24 bc	0.20 ± 0.01 c 0.23 ± 0.01 b 0.25 ± 0.02 a 0.22 ± 0.01 b	0.94 ± 0.02 de 1.07 ± 0.05 c 1.22 ± 0.04 b 1.03 ± 0.04 cd

Control (CK) and salt-treated (S) plants were sprayed with distilled water, while S + MT50, S + MT100, S + MT200, and S + MT500 plants were sprayed with 50, 100, 200, and 500 μM MT, respectively. Different lowercase letters indicate significant differences at the *p* ≤ 0.05 level

The soil and plant analyzer development (SPAD) value increased gradually over time (Table 2). The SPAD value under the salt treatment decreased significantly, and the SPAD value at 3, 6, 9, and 12 d decreased by 12%, 16%, 15%, and 18%, respectively, compared with the CK treatment. Under the 50 μM MT treatment, the SPAD value was higher than that of the S plants throughout the treatment period, but there was no significant difference. Under the 100 and 500 μM MT treatments, the SPAD value was significantly higher than that of the S plants at 3 and 6 d, with the most obvious increase under the 200 μM MT treatment. The SPAD value at 3, 6, 9, and 12 d increased by 10%, 14%, 9%, and 16%, respectively, compared with the S treatment. These differences were significant, indicating that treatment with 200 μM MT effectively alleviates the degradation of chlorophyll and promotes the synthesis of chlorophyll.

**Table 2:** Effects of melatonin on relative chlorophyll content of cotton seedling under salt stress

Treatment	0d	3d	6d	9d	12d
CK	37.10 ± 1.03 a	43.10 ± 1.53 a	47.22 ± 2.36 a	49.30 ± 2.4 a	49.30 ± 0.85 a
S	37.20 ± 1.40 a	38.04 ± 1.46 d	39.90 ± 1.73 d	42.10 ± 2.22 c	40.20 ± 1.81 c
S + MT50 S + MT100 S + MT200 S + MT500	37.02 ± 1.45 a 37.10 ± 1.10 a 37.10 ± 1.12 a 37.26 ± 1.14 a	38.70 ± 0.69 cd 40.22 ± 0.74 bc 41.70 ± 1.48 ab 39.84 ± 1.01 bc	41.90 ± 1.04 cd 43.50 ± 1.09 bc 45.34 ± 1.45 ab 42.90 ± 1.96 bc	43.32 ± 1.78bc 45.02 ± 1.7 bc 46.02 ± 1.82 b 44.46 ± 1.12bc	44.12 ± 1.48 c 45.22 + 1.71 bc 46.82 + 1.56 b 44.84 + 1.33 bc

Control (CK) and salt-treated (S) plants were sprayed with distilled water, while S + MT50, S + MT100, S + MT200, and S + MT500 plants were sprayed with 50, 100, 200, and 500 μM MT, respectively. Different lowercase letters indicate significant differences at the *p* ≤ 0.05 level

### Exogenous melatonin regulates the photosynthetic characteristics of cotton seedlings under salt stress

**Fig. 1:**
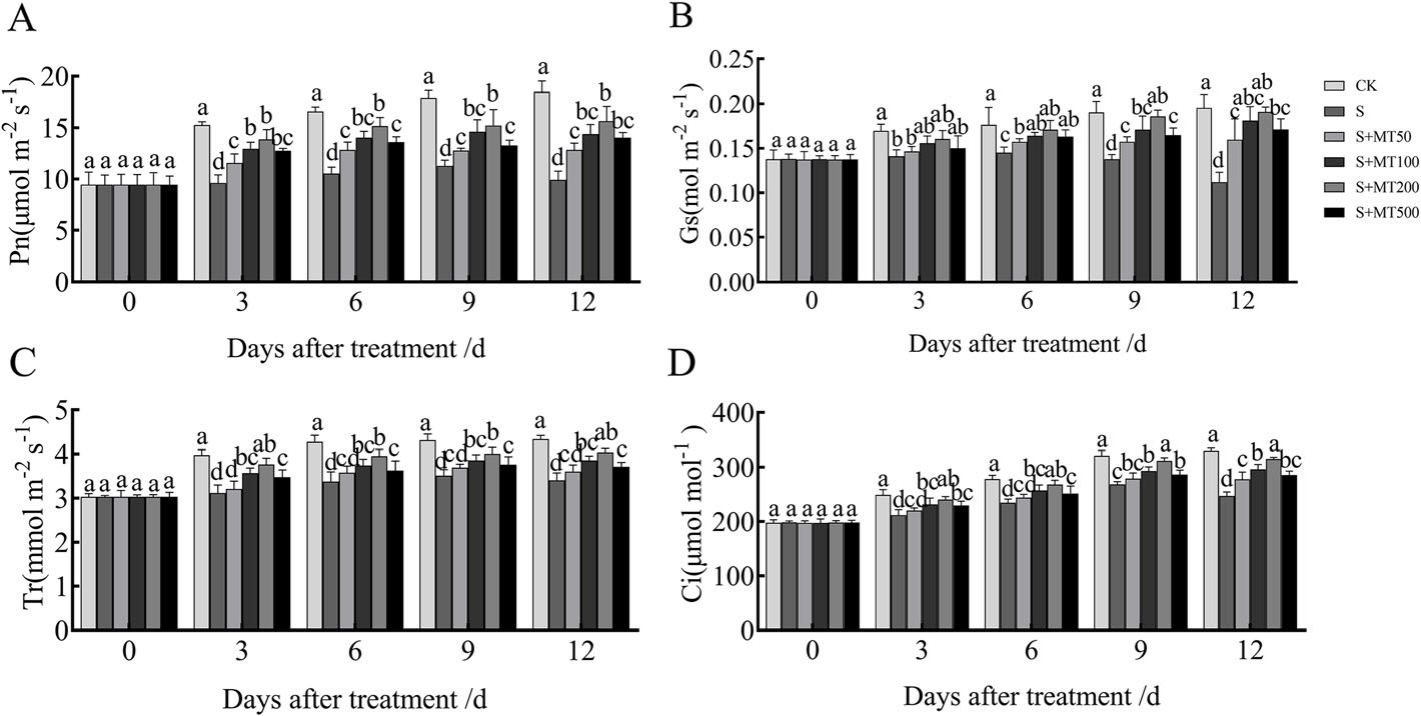
Effects of melatonin on gas-exchange parameters of cotton seedling under salt stress. Control (CK) and salt-treated (S) plants were sprayed with distilled water, while S + MT50, S + MT100, S + MT200, and S + MT500 plants were sprayed with 50, 00, 200, and 500 μM MT, respectively. Different lowercase letters indicate significant differences at the *p* ≤ 0.05 level

Figure 1A–D shows that the trends in the net photosynthetic rate (Pn), stomatal conductance (Gs), transpiration rate (Tr), and intercellular CO_2_ concentration (Ci) of the leaves were markedly similar and gradually increased over time.

The Pn under the S treatment decreased significantly, and the Pn at 3, 6, 9, and 12 d decreased by 40%, 36%, 37%, and 46%, respectively, compared with the CK plants (Fig. 1A). The Pn of cotton leaves increased the most significantly following treatment with 200 μM MT, and the Pn at 3, 6, 9, and 12 d increased by 44%, 44%, 35%, and 58%, respectively.

The Gs, Tr, and Ci under the S treatment decreased significantly, compared with the CK treatment (Fig. 1B–D). When treated with MT, they were significantly higher than that of the S treatment, and 200 μM MT had the greatest effect on promoting photosynthetic parameters. The Gs, Tr, and Ci at 12 d increased by 58%, 71%, 19%, and 27%, respectively, compared with the S treatment.

In summary, the treatment with 200 μM MT effectively alleviated the degradation of cotton chlorophyll and improved the photosynthetic capacity.

### Exogenous melatonin regulates the fluorescence characteristics of cotton seedlings under salt stress

**Fig. 2:**
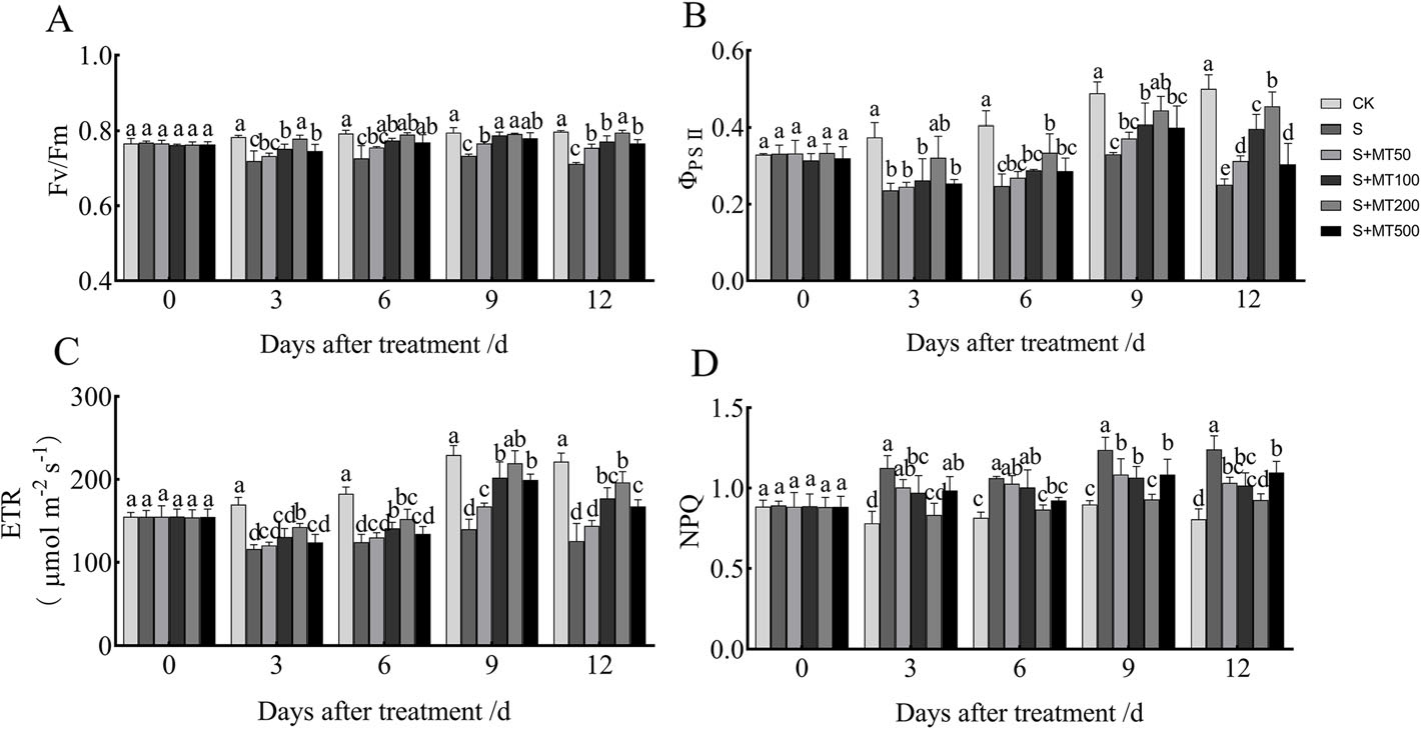
Effects of melatonin on chlorophyll fluorescence parameters of cotton seedling under salt stress. Control (CK) and salt-treated (S) plants were sprayed with distilled water, while S + MT50, S + MT100, S + MT200, and S + MT500 plants were sprayed with 50, 100, 200, and 500 μM MT, respectively. Different lowercase letters indicate significant differences at the *p* ≤ 0.05 level

The maximum photochemical efficiency of PSII (*Fv/Fm*) represents the original maximum fluorescence efficiency of PSII in the photosystem, which can reflect the efficiency of the photoreaction center PSII at converting light energy into chemical energy. The *Fv/Fm* under the S treatment decreased significantly, and the *Fv/Fm* at 3, 6, 9, and 12 d decreased by 8%, 8%, 8%, and 11%, respectively, compared with the CK plants (Fig. 2A). When treated with MT, the *Fv/Fm* was significantly higher than that of the S plants. The *Fv/Fm* of cotton leaves increased the most significantly following treatment with 200 μM MT, and the *Fv/Fm* at 3, 6, 9, and 12 d increased by 8%, 9%, 8%, and 12%, respectively.

PSII actual photochemical quantum yield (ΦPS II), and apparent electron transfer efficiency (ETR) under the S treatment decreased significantly, compared with the CK treatment (Fig. 2B, C). When treated with MT, they were significantly higher than that of the S treatment. Under 200 μM MT treatments, ΦPS II, and ETR were significantly higher than that of the S treatment at 12 d increased by 12%, 81%, and 57%, respectively, compared with the S treatment.

Non-photochemical quenching (NPQ) is the quenching of fluorescence caused by heat dissipation, which reflects the ability of plants to dissipate excess light energy into heat, known as its photoprotective ability. Compared with the CK treatment, the NPQ under the S treatment increased significantly (Fig. 2D). When treated with MT, the NPQ was significantly lower than that of the S treatment. The NPQ of cotton leaves decreased the most significantly following treatment with 200 μM MT, and the NPQ at 3, 6, 9, and 12 d decreased by 26%, 19%, 25%, and 25%, respectively.

This data indicates that treatment with 200 μM MT can effectively increase *Fv/Fm*, ΦPS II and ETR, reduce NPQ, and alleviate the damaging effect of salt stress on PSII of cotton leaves.

### Melatonin enhanced the content of proline in cotton seedlings under salt stress

**Fig. 3:**
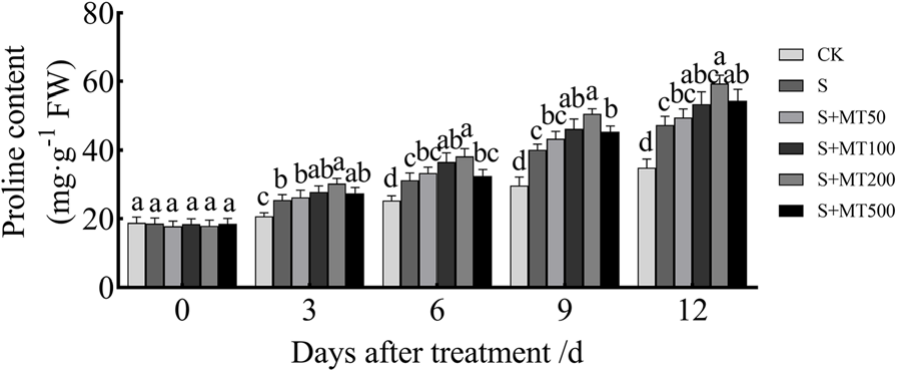
Effects of exogenous melatonin treatment on proline content of cotton seedlings under salt stress. Control (CK) and salt-treated (S) plants were sprayed with distilled water, while S + MT50, S + MT100, S + MT200, and S + MT500 plants were sprayed with 50, 100, 200, and 500 μM MT, respectively. Different lowercase letters indicate significant differences at the *p* ≤ 0.05 level

With the extension of stress time, the proline (pro) content of cotton leaves under the CK treatment and S both showed an upward trend. Compared with the CK treatment, the proline content increased significantly, by 23%, 24%, 35%, and 36% at 3, 6, 9, and 12 d, respectively, following the S treatment (Fig. 3). When treated with MT, the proline content was significantly higher than that of the S treatment. The proline content of cotton leaves increased most significantly following treatment with 200 μM MT, and the proline content at 3, 6, 9, and 12 d increased by 19%, 22%, 26%, and 25%, respectively.

### Exogenous melatonin improves ionic homeostasis under salt stress

**Fig. 4:**
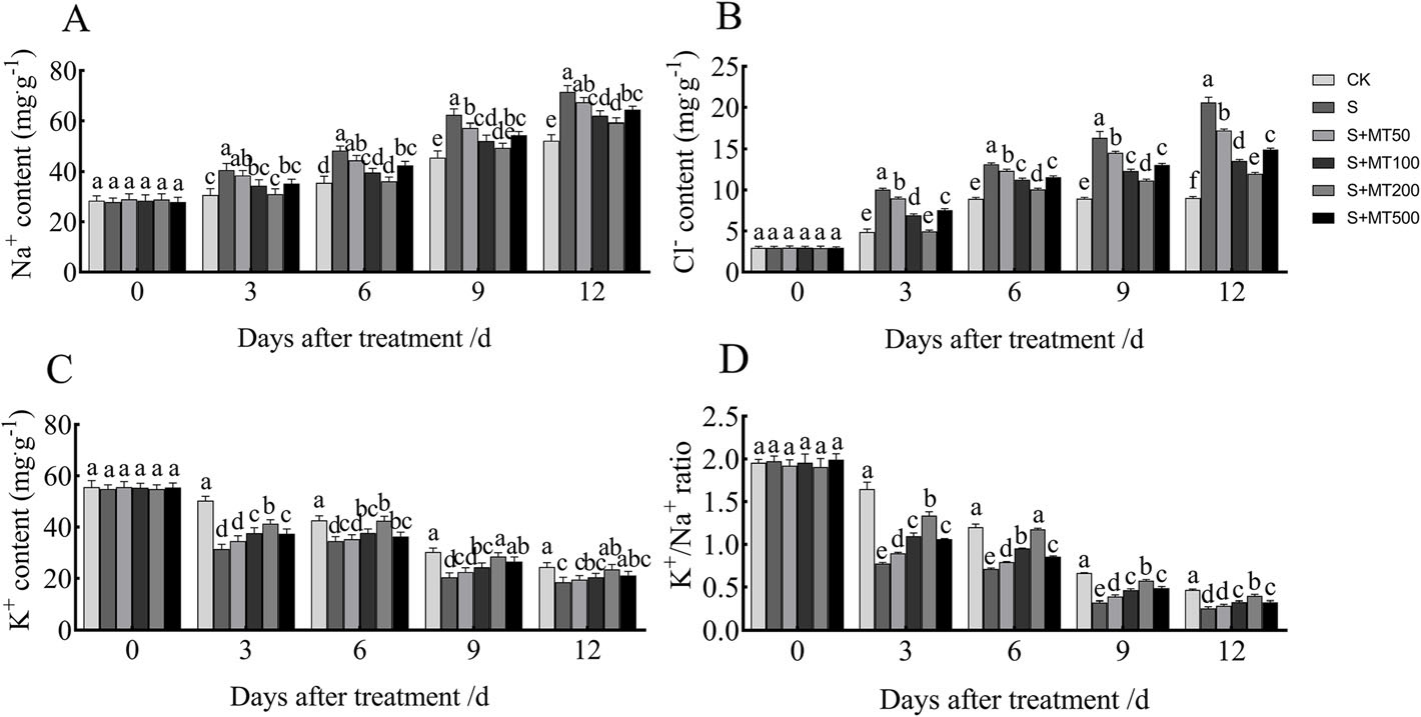
Effects of exogenous melatonin treatment on Na^+^ (A), Cl^−^ (B), K^+^ (C) content and K^+^/Na^+^ ratio (D) of cotton seedlings under salt stress. Control (CK) and salt-treated (S) plants were sprayed with distilled water, while S + MT50, S + MT100, S + MT200, and S + MT500 plants were sprayed with 50, 100, 200, and 500 μM MT, respectively. Different lowercase letters indicate significant differences at the *p* ≤ 0.05 level

Plants will accumulate Na^+^ under salt stress, which results in damage to them. Figure 4A, B shows that the trends in contents of Na^+^ and Cl^**−**^ of leaves were readily apparent and increased gradually over time. However, the content of K^+^ and ratio of K^+^/Na^+^ decreased gradually over time, reaching their lowest values at 12 d (Fig. 4C, D).

Compared with the CK treatment, the content of Na^+^ under the S treatment increased significantly, by 32%, 36%, 37%, and 37% at 3, 6, 9, and 12 d, respectively (Fig. 4A). When treated with MT treatments, the content of Na^+^ was significantly lower than that of the S plants. The Na^+^ content of cotton leaves decreased the most significantly following treatment with 200 μM MT, and the content of Na^+^ at 3, 6, 9, and 12 d decreased by 24%, 25%, 21%, and 17%, respectively. The change trend of Cl^**−**^ and Na^+^ was basically the same (Fig. 4B). These results indicate that 200 μM MT prevents harmful ions from entering the cell to some extent, thereby effectively protecting the cell structure.

Compared with the CK treatment, the K^+^ content under the S treatment decreased significantly by 37%, 19%, 33%, and 24% at 3, 6, 9, and 12 d, respectively (Fig. 4C). Following treatment with MT, the K^+^ content was significantly higher than that of the S plants. The K^+^ content of cotton leaves increased the most significantly following treatment with 200 μM MT, and the K^+^ content at 3, 6, 9, and 12 d increased by 31%, 23%, 40%, and 27%, respectively. Similarly, the change trend of K^+^/Na^+^ and K^+^ was consistent (Fig. 4D). These results indicate that 200 μM MT had the most notable effect on increasing the content of K^+^ and K^+^/Na^+^ ratio of cotton leaves.

### Histochemical analyses of the contents of ROS and fluorescence microscopy in cotton leaves

**Fig. 5:**
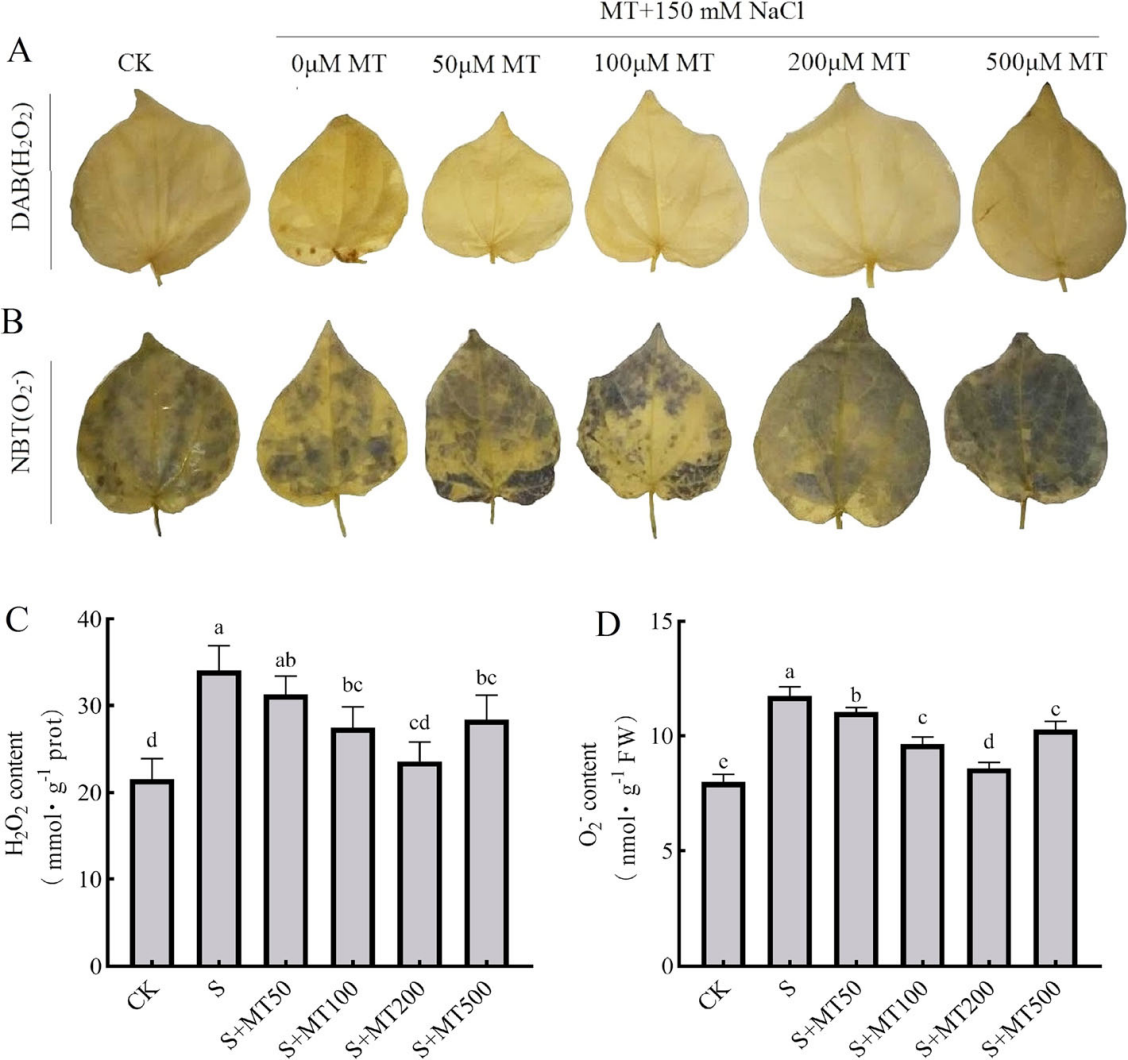
The effect of melatonin on the generation H_2_O_2_ and ${\mathrm{O}}_2^{\cdotp -}$ in the leaves of cotton seedlings under salt stress. The leaves of seedlings were treated with control (CK), salt-treated (S), S + MT50, S + MT100, S + MT200, and S + MT500 for 72 h. After treatment, the leaves were excised and supplied DAB or NBT for 7 h. Control (CK) and salt-treated (S) plants were sprayed with distilled water, while S + MT50, S + MT100, S + MT200, and S + MT500 plants were sprayed with 50, 100, 200, and 500 μM MT, respectively. Different lowercase letters indicate significant differences at the *p* ≤ 0.05 level

Figure 5A, B illustrates the histochemical detection of the contents of hydrogen peroxide (H_2_O_2_) and superoxide anions (${\mathrm{O}}_2^{\cdotp -}$) in the leaves of cotton plants. Under salt stress, H_2_O_2_ was detected using DAB for the formation of brown spots, and ${\mathrm{O}}_2^{\cdotp -}$ was detected using NBT staining for the formation blue color, which respectively spread over almost all of the leaf area. It can be seen from the histochemical staining that the treatment with MT under salt stress noticeably reduced the content of H_2_O_2_ and ${\mathrm{O}}_2^{\cdotp -}$. Among them, the brown spots and blue areas of cotton leaves treated with MT were the lowest. Compared with the CK treatment, the contents of H_2_O_2_ and ${\mathrm{O}}_2^{\cdotp -}$ under the S treatment increased significantly by 58% and 47%, respectively (Fig. 5C, D). The contents of H_2_O_2_ and ${\mathrm{O}}_2^{\cdotp -}$ of cotton leaves decreased the most significantly following treatment with 200 μM MT, and the contents of H_2_O_2_ and ${\mathrm{O}}_2^{\cdotp -}$ decreased by 31% and 27%, respectively, indicating that 200 μM MT had the most obvious effect on removing ROS from cotton leaves. These results suggest that 200 μM MT was able to alleviate the accumulation of ROS in cotton seedlings under salt stress.

**Fig. 6:**
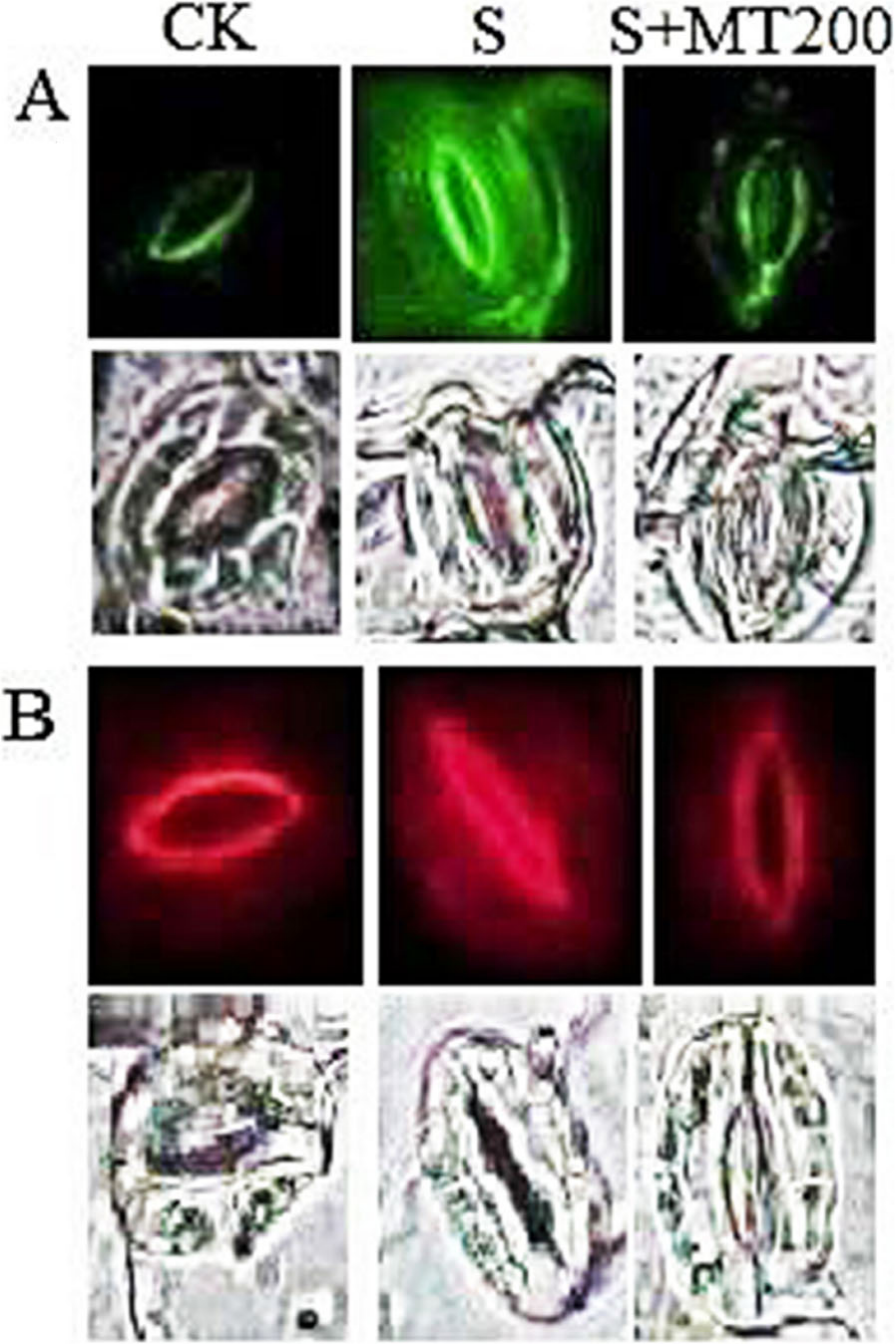
Representative fluorescence microscopic images of in vivo detection of hydrogen peroxide (H_2_O_2_), superoxide anion (${\mathrm{O}}_2^{\cdotp -}$) in leaves of cotton plants treated with different chemical compounds. (**A**) H_2_O_2_ detected with the DCF-DA fluorescence probe (green color) and its corresponding bright field (lower panels). (**B**) ${\mathrm{O}}_2^{\cdotp -}$ detected with 10 μM DHE (red color) and its corresponding bright field (lower panels). The leaves of seedlings were treated with control (CK), salt-treated (S), S + MT200 for 72 h. Control (CK) and salt-treated (S) plants were sprayed with distilled water, while S + MT200 was sprayed with 200 μM MT

To further observe the effect of MT on the accumulation of ROS, we observed the structure of stomatal guard cells under three treatments (CK, S, S + MT200). Figure 6A, B illustrates the fluorescence detection of the contents of both ROS in guard cells. Compared with the CK treatment, the content of H_2_O_2_ (green fluorescence) and ${\mathrm{O}}_2^{\cdotp -}$ (red fluorescence) detected in the guard cells increased substantially under salt stress. However, the contents of H_2_O_2_ and ${\mathrm{O}}_2^{\cdotp -}$ in the cotton guard cells treated with exogenous 200 μM MT were significantly reduced.

### Exogenous melatonin facilitates stomatal opening

**Fig. 7:**
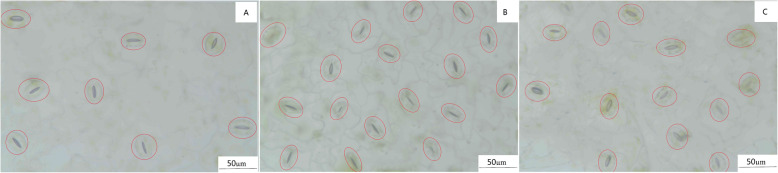
The effect of melatonin on the stomatal characteristics under salt stress. (**A**): CK. (**B**): S. (**C**): S + MT200. Scale bars = 50 μm. The leaves of seedlings were treated with control (CK), salt-treated (S), S+MT200 for 12 d. Control (CK) and salt-treated (S) plants were sprayed with distilled water, while S + MT200 was sprayed with 200 μM MT

Stomata are important channels for the exchange of water vapor and air in plant leaves and are extremely important for the accumulation of organic material. Salt stress significantly reduced the stomatal length (17%), width (22%), aperture (SA) (42%), and increased the number 93%) compared with the CK treatment (Table 3; Fig. 7A, B). However, the stomatal length, width and aperture increased by 10%, 16%, and 96%, respectively, and the stomatal number (SN) decreased by 23% after treatment with MT compared with the salt treatment (Table 3; Fig. 7B, C).

**Table 3:** Effects of exogenous melatonin on the stomatal characteristics under salt stress.

Treatment	Stomatal length (μm)	Stomatal width (μm)	Stomatal aperture (μm)	Stomatal number
CK	32.49 ± 1.68 a	21.64 ± 1.33 a	4.39 ± 0.84 a	8.40 ± 0.49 c
S	27.09 ± 1.49 c	16.98 ± 0.95 c	2.54 ± 0.25 b	16.20 ± 1.17 a
S + MT200	29.72 ± 1.13 b	19.64 ± 1.09 b	4.98 ± 0.32 a	12.40 ± 0.49 b

The stomata length, width, pore diameter and number of cotton seedlings under different treatments. Control (CK) and salt-treated (S) plants were sprayed with distilled water, while S + MT200 was sprayed with 200 μM MT. Different lowercase letters indicate significant differences at the *p* ≤ 0.05 level

### Effects of melatonin on ultrastructural alterations in mesophyll cells and the chloroplast of cotton leaves under salt stress

**Fig. 8:**
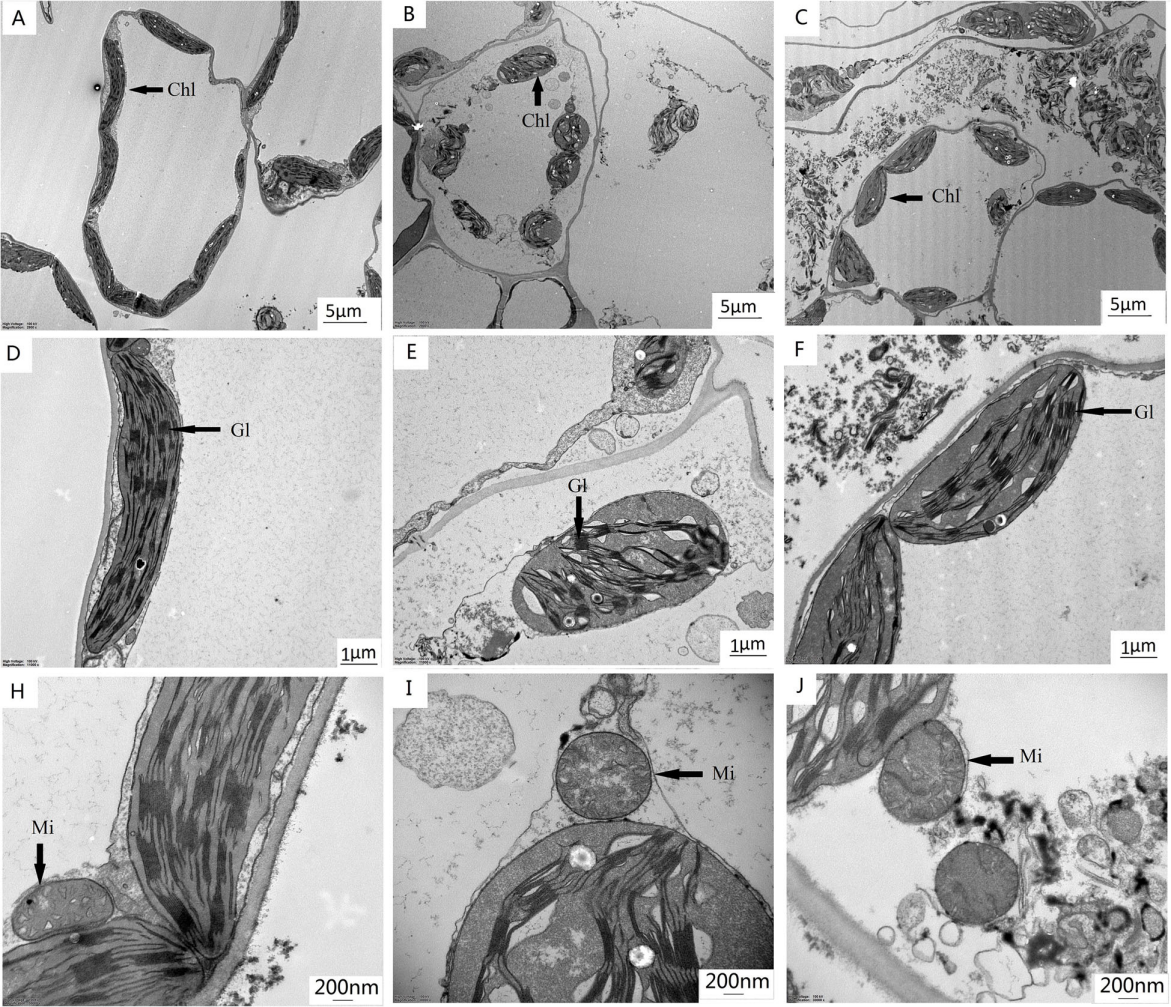
Transmission electron microscopy (TEM) of mesophyll cells of cotton leaf. (**A**–**C**) figure shows the TEM structure of mesophyll cell of CK, S, and S + MT200 plants, respectively; (**D**–**F**) figure shows the relatively high magnified view of chloroplasts of the plant of CK, S, and S + MT200 plants, respectively; (**H**–**J**) figure shows the relatively high magnified view of mitochondria of the plant of CK, S, and S + MT200 plants, respectively. Control (CK) and salt-treated (S) plants were sprayed with distilled water, while S + MT200 was sprayed with 200 μM MT. *Chl* chloroplast, *Mi* mitochondrion, *Gl* Grana lamellae

Different sizes of cells and chloroplast structures were apparent in an examination of the ultrastructure of the whole mesophyll and chloroplasts of the third functional leaf treated with salt for 12 days, the control, salt, and 200 μM MT-treated plants (Fig. 8). In the CK treatment, the chloroplasts were elliptical, neatly arranged, and closely attached to the cell wall (Fig. 8A, D). Compared with the CK treatment, the shape of the mesophyll cells of the plant leaves under salt stress was not significantly deformed, but the mesophyll cell structure was destroyed (Fig. 8B). In addition, compared with CK, the length of the chloroplasts in the leaves under salt stress decreased; the width increased; the aspect ratio decreased; the number of basal grains in the chloroplasts decreased, and the lamella structure was disordered (Fig. 8D, E). Alternatively, the TEM (transmission electron microscopy) micrographs of the plant leaves treated with 200 μM MT under salt stress indicate that the mesophyll cells of the leaves did not have the phenomenon of plasmoplasmic wall separation; the chloroplasts were regular in shape and close to the cell wall; the lamella structure was complete; the layers were clear, and the arrangement was neat (Fig. 8C, F). In addition, the mitochondria will be partially decomposed under salt stress, but the mitochondrial structure of cotton treated with MT under salt stress did not change significantly compared with the CK (Fig. 8H–J). Therefore, compared with the salt-treated plants, the 200 μM MT treatment in the salt-stressed plants significantly alleviated the salt stress-induced oxidative damage and repaired the complete cell and chloroplast structure.

### Melatonin treatment improved the transportation system of xylem vessels

**Fig. 9:**
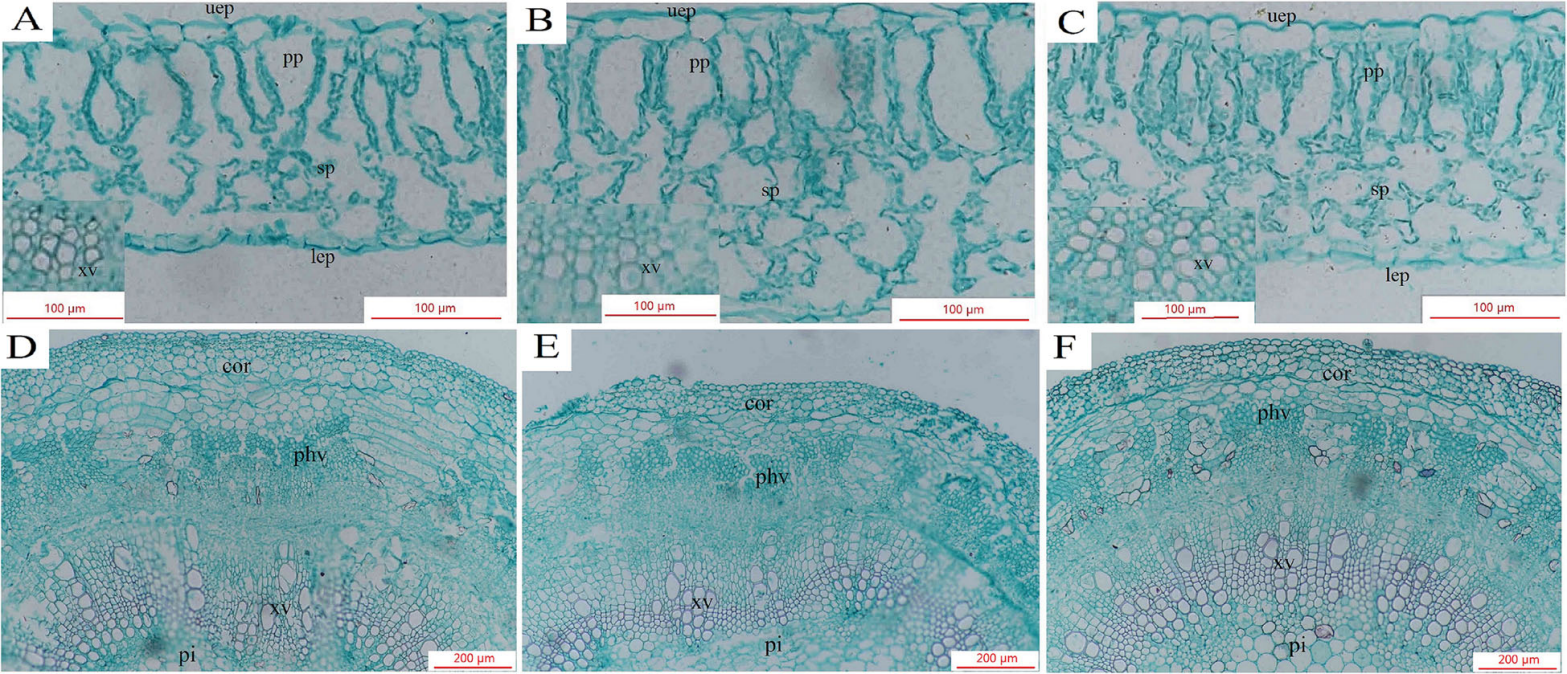
Transections of cotton seedling leaf blade and stem affected by the application of melatonin and salt stress. (**A**, **D**): CK. (**B**, **E**): S. (**C**, **F**): S + MT200. Control (CK) and salt-treated (S) plants were sprayed with distilled water, while S + MT200 was sprayed with 200 μM MT. *Uep* upper epidermis, *pp* plastid parenchyma, *sp* sponge parenchyma, *lep* lower epidermis, *xv* xylem vessels, *phv* phloem vessels, *cor* cortex, *pi* pith. Bars in the rectangular cross section = 100 μm

The stems and leaves of cotton seedlings treated with normal control (CK), salt (S) and salt stress + 200 μM MT (S + MT200) for 12 days were dissected and observed. As shown in Table 4, the thickness of the blade, palisade (PT), and spongy (ST), area of the xylem vessel (LAX), area of the phloem vessel (LAP), and the upper and down surface thickness all increased in response to salt stress. Compared with the S treatment, the thickness of the blade, palisade, and spongy parenchyma, and the upper and down surface thickness decreased with the MT treatment. However, the MT treatment had a high impact on the area of leaf xylem and phloem vessels, as shown in Fig. 9A–C. Seedlings treated with 200 μM MT exhibited positive changes in these characteristics in the presence of the salt stress and maintained the leaf anatomical characteristics at the status in which plants could overcome salt stress.

**Table 4:** Effects of exogenous melatonin on mesophyll and vascular bundle anatomical structure in cotton seeding under salt stress

Treatment	CK	S	S + MT200
Leaf thickness (um)	168.33 ± 9.95 c	241.43 ± 9.58 a	189.75 ± 3.43 b
Palisade tissue thickness (um)	67.46 ± 3.75 c	96.23 ± 3.98 a	79.22 ± 1.26 b
Spongy tissue thickness (um)	69.80 ± 3.58 b	113.43 ± 3.10 a	73.73 ± 4.16 b
Palisade/Spongy	0.88 ± 0.09 b	0.97 ± 0.10 b	1.14 ± 0.038 a
Leaf structure tightness	0.37 ± 0.02 b	0.41 ± 0.05 ab	0.43 ± 0.01 a
Leaf structure looseness	0.41 ± 0.004 b	0.46 ± 0.016 a	0.41 ± 0.008 b
Upper surface thickness (um)	14.33 ± 1.55 c	22.67 ± 1.11 a	19.75 ± 0.54 b
Down surface thickness (um)	12.50 ± 0.47 c	16.26 ± 0.70 a	13.75 ± 0.80 b
Xylem thickness (um)	56.44 ± 1.09 c	67.44 ± 2.90 b	91.17 ± 3.10 a
Phloem thickness (um)	57.31 ± 1.51 c	69.49 ± 5.68 b	81.98 ± 6.02 a
Area of a vessel (um^2^)	220.71 ± 11.56 c	263.40 ± 9.25 b	413.46 ± 10.41 a
Area of xylem (10^4^ * um^2^)	0.46 ± 0.03 c	0.90 ± 0.08 b	1.55 ± 0.03 a
Area of phloem (10^4^ * um^2^)	0.91 ± 0.03 c	1.70 ± 0.17 b	2.44 ± 0.13 a

Control (CK) and salt-treated (S) plants were sprayed with distilled water, while S + MT200 was sprayed with 200 μM MT. Different lowercase letters indicate significant differences at the *p* ≤ 0.05 level

The diameter of the stem under salt stress decreased significantly compared with the CK (Table 5; Fig. 9D–F). This was primarily because of the thickness of the pith diameter, the xylem vessel (SXT), and the phloem vessel (SPT) decreased by 12%, 7%, and 13%, respectively. Seedlings treated with 200 μM MT significantly improved the stem anatomical characteristics, such as the pith diameter, thickness of the xylem vessel and thickness of the phloem vessel increased by 8%, 6%, and 7%, respectively.

**Table 5:** Effects of exogenous melatonin on the anatomical structure of cotton stems under salt stress.

Treatment	Xylem thickness (um)	Phloem thickness (um)	Epidermis thickness (um)	Pith diameter (um)
CK	291.86 ± 2.58 a	193.93 ± 2.23 a	11.80 ± 0.22 a	1304.92 ± 13.23 a
S	271.98 ± 1.48 b	168.65 ± 2.78 c	11.71 ± 0.21 a	1142.96 ± 14.47 c
S + MT200	289.48 ± 4.52 a	181.08 ± 2.58 b	11.79 ± 0.12 a	1236.88 ± 5.46 b

Control (CK) and salt-treated (S) plants were sprayed with distilled water, while S + MT200 was sprayed with 200 μM MT. Different lowercase letters indicate significant differences at the *p* ≤ 0.05 level

### Aboveground trait correlations and variations

**Fig. 10:**
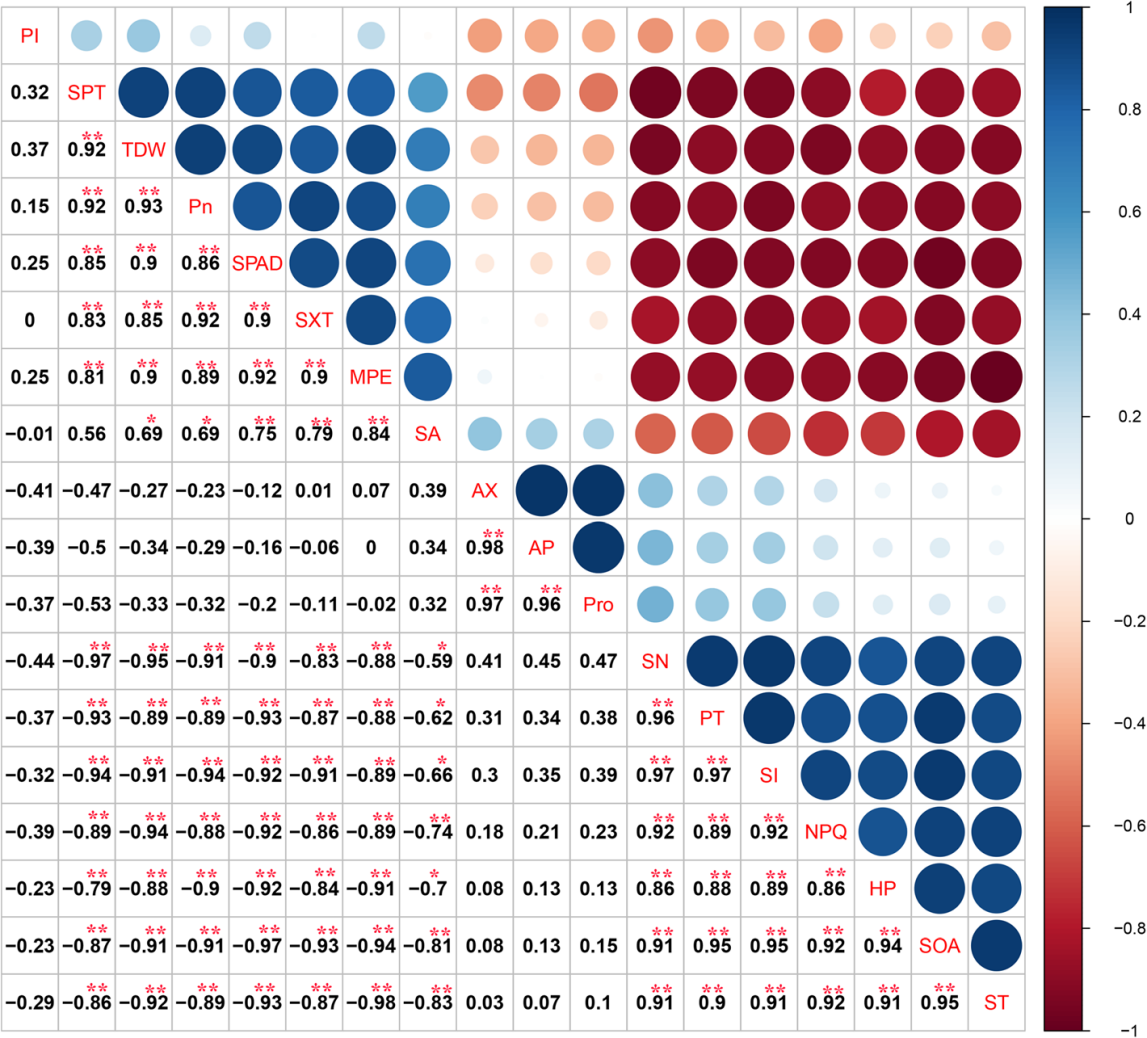
The matrix of the Spearman’s correlation coefficients and 95% confidential circle among aboveground traits. The significance level of correlations is indicated; * *p* < 0.05; ** *p* < 0.01. From left to right, abbreviations stand for: *K*^*+*^ potassium ion, *SPT* stem phloem thickness, *TDW* total dry weight, *Pn* the net photosynthetic rate, *SPAD* SPAD value, *SXT* stem xylem thickness, *Fv/Fm* the maximum photochemical efficiency of PSII, *SA* stomatal aperture, *LAX* leaf area of xylem, *LAP* leaf area of phloem, *Pro* proline, *SN* stomatal number, *PT* palisade tissue thickness, *Na*^*+*^ sodium ion, *NPQ* non-photochemical quenching, *H*_*2*_*O*_*2*_ hydrogen peroxide, ${O}_2^{\cdotp -}$ superoxide anion, *ST* spongy tissue thickness

To more effectively identify the correlation between these traits, a Spearman’s correlation analysis was conducted for 18 representative traits. As indicated in Fig. 10, the TDW significantly positively correlated with the Pn, and both significantly positively correlated with the SPAD, *Fv/Fm*, SA, SXT and SPT but significantly negatively correlated with the SN, PT, Na^+^, NPQ, H_2_O_2_, ${\mathrm{O}}_2^{\cdotp -}$ and ST. The TDW, Pn, SPAD, *Fv/Fm*, SA significantly negatively correlated with the SN, PT, Na^+^, NPQ and ST. H_2_O_2_ significantly positively correlated with ${\mathrm{O}}_2^{\cdotp -}$, and both were negatively correlated with the Pn, SPAD, and TDW.

**Fig. 11:**
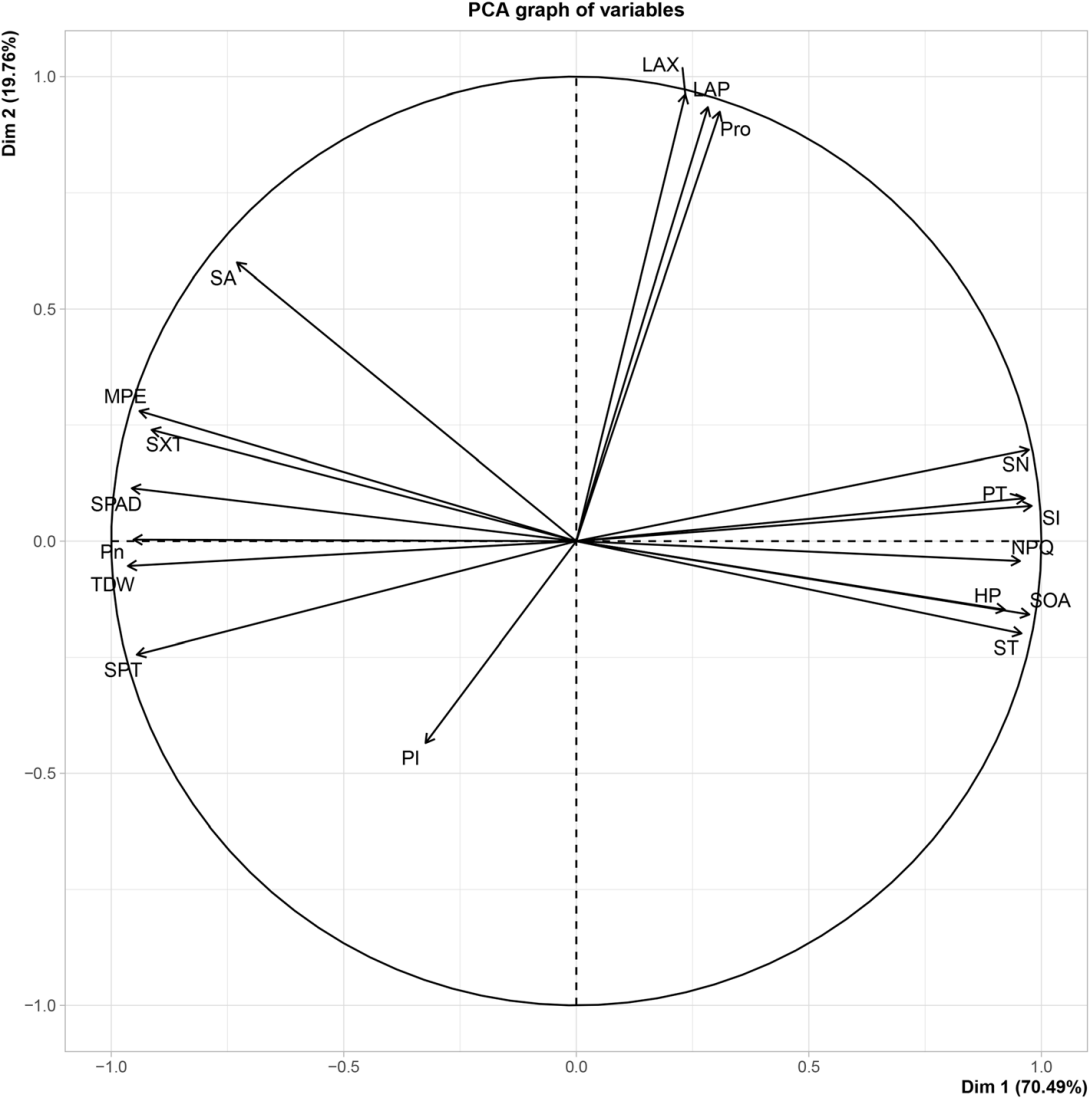
Principal component analysis (PCA) of 18 aboveground traits. Clockwise, the abbreviations stand for: *LAX* leaf area of xylem, *LAP* leaf area of phloem, *Pro* proline, *SN* stomatal number, *PT* palisade tissue thickness, *Na*^*+*^ sodium ion, *NPQ* non-photochemical quenching, *H*_*2*_*O*_*2*_ hydrogen peroxide, ${O}_2^{\cdotp -}$ superoxide anion, *ST* spongy tissue thickness, *K*^*+*^ potassium ion, *SPT* stem phloem thickness, *TDW* total dry weight, *Pn* the net photosynthetic rate, *SPAD* SPAD value, *SXT* stem xylem thickness, *Fv/Fm* the maximum photochemical efficiency of PSII, *SA* stomatal aperture

Subsequently, a principal component analysis was performed for 18 representative traits. TDW, Pn, SPAD, and PT, Na^+^, NPQ, H_2_O_2_, and ${\mathrm{O}}_2^{\cdotp -}$ were almost fully loaded on the PCA axes 1 but in an opposite direction (Fig. 11). The first dimension explains 70.49% of the total variation. LAX, LAP, and pro were loaded on the PCA axes 2. The second dimension explains 19.76% of the total variation. Together, two dimensions explained 90.25% of the total variation of all 18 in aboveground traits.

## Discussion

In this experiment, the cotton seedling biomass, photosynthetic fluorescence, accumulation of osmotic substances, stomatal characteristics, ultrastructure and anatomical structure changes were studied by hydroponics cultivation of seedlings to attempt to illustrate the regulation of treatment with MT on cotton under salt stress. This study found that salt stress reduced the dry and fresh weights of the shoots and roots of cotton seedlings. Treatment with MT can significantly increase the biomass of shoots and roots and promote the growth of cotton seedlings (Table 1). Similar studies have confirmed that MT can promote plant growth in tomato and cucumber [51, 52].

Chloroplast chlorophyll participates in the absorption and transmission of light energy during photosynthesis. It is an important substance for plant photosynthesis to capture light energy, and its content is not only an important indicator of plant physiological characteristics but is also the basis of photosynthesis [1]. Chlorophyll is easily degraded, and its synthesis is inhibited under salt stress [53]. Treatment with MT significantly increased the Pn of seedlings under abiotic stress conditions, alleviated the degradation of chlorophyll, and improved the drought resistance of seedlings [54, 55]. This study found that salt stress reduced the chlorophyll content, Pn, Gs, and Ci of cotton seedlings (Table 2; Fig. 1), indicating that the decrease in Pn of cotton seedling leaves was primarily the result of stomatal limitation. Exogenous MT can significantly enhance the Pn of cotton and increase the Pn and Ci. In addition, there was some degree of dose relationship between the enhancement effect and concentration of MT. The effect was the most apparent following the treatment with 200 μmol/L MT. This is consistent with the results of Huang et al. [37] on the photosynthetic capacity of maize under drought conditions alleviated by MT. This indicates that MT can protect the chloroplast structure and stability of photosynthetic organs to some extent, maintain a high level of chlorophyll, enhance the ability of leaves to capture light energy, promote photosynthesis, and effectively alleviate the inhibitory effect of leaf photosynthesis under salt stress.

The reaction centers of plant photosynthesis are photosystem II and photosystem I. Photosystem II is sensitive to adversity stress. The chlorophyll fluorescence parameter is an effective index that reflects the photosynthetic electron transfer rate and light energy utilization rate of reaction center PS II [56]. The study found that the *Fv/Fm* of plant seedlings decreased under salt stress, and exogenous treatment with MT increased the *Fv/Fm* in the seedlings and improved the salt tolerance of tomato, wheat, and tea [46, 57, 58]. The NPQ of apple seedlings increased under salt stress, but the NPQ of seedlings treated with exogenous MT was significantly reduced [29]. This study found that salt stress reduced the *Fv/Fm*, ETR, and PS II of cotton seedlings and increased the NPQ. Exogenous MT can significantly enhance the *Fv/Fm*, ETR, and PS II of plants, reducing NPQ, and 200 μM MT had the best treatment effect (Fig. 2), which is consistent with those of Hu et al. [59] on the photosynthesis of Bermuda grass following treatment with MT to alleviate low temperatures. This is because the external application of MT can effectively inhibit the potential decline of PS II activity under salt stress and protect the photosynthetic system to some extent. This effect enables the light energy absorbed by leaves to be used for photosynthetic electron transfer to the greatest extent, so as to alleviate the inhibitory effect of salt stress on electron transfer rate and light energy conversion efficiency.

Plants will increase the synthesis and accumulation of osmotic adjustment substances (proline, soluble protein, soluble sugar, and betaine) under adversity stress and the response to adversity stress [60]. Among them, proline is a small molecular substance that adjusts the osmotic content of cells. This compound can stabilize macromolecules and cell walls and protect hydrophobic enzymes. The content of proline will increase significantly under adversity stress [32]. Treatment with MT significantly increases the content of proline, polyamines, and sucrose, up-regulates the expression of proline biosynthesis genes (*P5CS*) in tomato seedlings under temperature stress, and alleviate the damage of temperature stress on tomato plants [51, 61]. In this experiment, with the extension of treatment time, the content of proline in cotton leaves continued to increase under the control and NaCl treatments. Under salt stress, the proline content in cotton leaves increased significantly, but after spraying exogenous MT, the proline content in cotton leaves increased significantly (Fig. 3). This is consistent with the results of Meng et al. [44], in which MT relieves the osmotic stress of grapes under water stress, which indicates that the compound enhances the salt tolerance of cotton by increasing the content of proline.

Ion homeostasis is considered to be one of the most important mechanisms of plant responses to salt stress [62]. Under salt stress, ion homeostasis involves the basic feature of regulating living cells by adjusting the ion flux, which is critical for the accumulation of essential ions such as K^+^ and the maintenance of a low concentration of toxic ions such as Na^+^ [63, 64]. Intracellular ion homeostasis is not only important for enzymes in the cytoplasm but also for the maintenance of membrane potential and the regulation of cell volume [63]. Under salt stress, the content of Na^+^ will accumulate in plants, reducing the content of K^+^ and the K^+^/Na^+^ ratio; treatment with MT increases the K^+^ content of crabapple and rapeseed and increases the K^+^/Na^+^ ratio [65–67], thereby increasing the salt tolerance of plants. In this experiment, the extension of the treatment time in control and NaCl treatments resulted in a continued increase in the content of Na^+^ in the cotton leaves and a continued decrease in the content of K^+^ in the cotton leaves. Under salt stress, the Na^+^ content in cotton leaves increased significantly, while the K^+^ content in cotton leaves decreased significantly. After spraying exogenous MT, the Na^+^ content in cotton leaves was significantly reduced, while the K^+^ content and K^+^/Na^+^ increased significantly (Fig. 4), which is consistent with the results of Castanares et al. [68] on the alleviation of ion toxicity of melon under salt stress following treatment with MT. This indicates that MT enhances the salt tolerance of cotton by reducing the accumulation of Na^+^ and increasing the absorption of K^+^ to maintain the K^+^/Na^+^ ratio. This study also confirmed that 200 μM MT helps maintain the ion balance under salt stress.

It has been found that excessive ROS produced under conditions of adversity can cause the oxidative damage of macromolecules (proteins, lipids, chlorophyll, and nucleotides), leading to cell death [69]. Therefore, it will cause changes in the content of some specific indicators, such as ROS (H_2_O_2_ and ${\mathrm{O}}_2^{\cdotp -}$) and malondialdehyde, which can be used as indicators of oxidative stress on plants. The results of this study show that treatments with different concentrations of MT can reduce the contents of H_2_O_2_ and ${\mathrm{O}}_2^{\cdotp -}$ in the leaves of cotton seedlings under salt stress to varying degrees (Figs. 5 and 6), and spraying 200 μM MT can more effectively reduce the oxidative damage caused by salt stress. It shows that exogenous MT can eliminate excessive ROS by promoting the activity of antioxidant enzymes to alleviate the oxidative stress caused by salt stress [50]. The mitigation of plant oxidative stress under stress by MT has been demonstrated in other plant species, such as oat [48], tea [46], rubber [70], tomato [57, 71], and watermelon [68].

The stomata are channels for the exchange of water and gas in the plant with the external environment, and the size, number and regulatory function of stomata are related to physiological processes such as transpiration [72]. Stomata are affected by many environmental factors, and salt stress reduces the level of CO_2_ in the mesophyll by causing changes in leaf photochemistry and carbon metabolism, which directly affect the photosynthesis of plants [8, 73]. Under salt stress, the length and width of the stomata and the width of the pore diameter of plant decrease, and the density of stomata increases [65]. The MT priming, spraying treatments and pretreatment with MT significantly improved the drought tolerance of two *Malus* species and rape seedlings, increased the length, width and opening of the stomata, and improved their function [60, 74, 75]. This study showed that cotton stomata were small and increased in number under salt stress treatment (Fig. 7), which may be owing to the restriction of salt stress on the expansion of leaf area. Small and dense stomata also have higher flexibility. MT treatment can increase the size and opening of the large pores and reduce the stomata density. It could be that MT promotes the extension of cotton leaves and reduces the stomatal density. This is consistent with the results of Meng et al. [44] on the alleviation of the photosynthetic capacity of grapes under water stress following treatment with MT.

The chloroplast is place where plant photosynthesis occurs, and it is also the main place where plant cells produce ROS and is the most sensitive to abiotic stress [76, 77]. Mitochondria are the main place where cells conduct the respiration that participates in cell growth and division through energy metabolism and is also one of the most sensitive organelles in plants to the external environment. Changes in the external environment will cause mitochondrial dysfunction and increase ROS and affect the activity of antioxidant enzymes and changes in the expression of key proteins [78]. Previous studies have also reported that MT alleviates the growth of tomato [79], maize [80, 81], wheat [42] and grapes [44] under adverse conditions, with less chloroplast damage, thicker leaves and an improvement in photosynthesis. In this study, the mesophyll cells and chloroplasts of cotton leaves under salt stress were significantly deformed, but treatment with MT could reduce the damage caused by salt stress (Fig. 8), which may be owing to the increase in intracellular proline and the elimination of excessive ROS by the antioxidant system, thereby reducing the damage of ROS to the plant cell membrane system.

The xylem and phloem are the main structures for water and nutrient transport in plants, and small stomatal openings and small xylem and phloem under stress conditions can significantly reduce the transport of water and nutrients from roots to the shoots [45, 82]. Studies on the anatomical structure of the leaves of zucchini under salt stress found that this type of stress increases the thickness of leaves, palisade tissue, sponge tissue, and intercellular spaces [83]. MT treatment can improve the stomatal characteristics of rapeseed seedlings and the anatomical structure of leaf stems under adversity conditions, protect the ultrastructure of chloroplasts, and increase the plant’s tolerance to stress [45, 74]. In this study, under salt stress, the diameter of the stem and the area of the vascular bundle of cotton seedlings was significantly reduced, and the leaf thickness significantly increased, which may be owing to the inhibition of cell expansion and elongation (Figs. 9 and 10; Tables 4 and 5). In addition, spraying MT on cotton seedlings under salt stress can improve the anatomical characteristics of the leaves and stems, particularly the stem diameter and the area of the xylem and phloem, thereby increasing the efficiency of water and nutrient transport and promoting the growth and development of cotton seedlings.

A correlation analysis of 18 representative indicators showed that the Pn significantly positively correlated with the TDW, SPAD, *Fv/Fm*, SA, SXT, and SPT and negatively correlated with the SN, PT, Na^+^, NPQ, H_2_O_2_, ${\mathrm{O}}_2^{\cdotp -}$, and ST. This is consistent with the results of Mohamed et al. [45] on the alleviation of photosynthetic inhibition of rapeseed under salt stress following treatment with MT. Interestingly, the Pn was negatively correlated with PT and ST (Fig. 11). We hypothesize that this correlation may be related to the number and location of chloroplasts, which merits further study.

## Conclusions

**Fig. 12:**
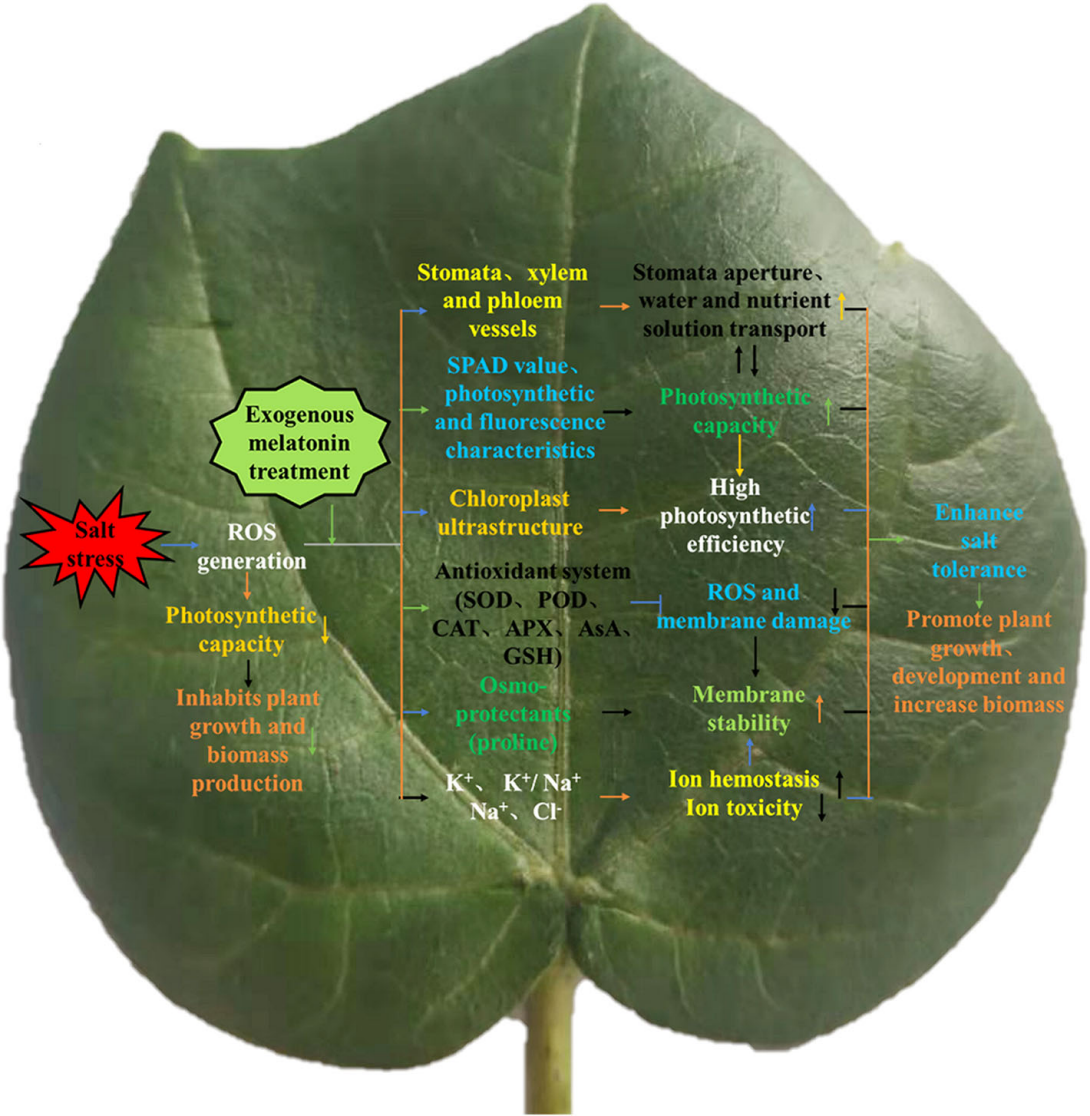
Mechanism model diagram of melatonin improving salt tolerance of cotton seedlings

To determine how MT mitigates the adverse effects of salt stress on cotton seedlings, we described a possible mechanism (Fig. 12). In this study, we confirmed that salt stress reduces the photosynthetic capacity of plants, and inhabits plant growth and biomass production. Cotton stomata will become smaller and increase in numbers under salt stress, but the stomata opening will become smaller, which limits the amount of gas exchange per unit area per unit time and reduces the amount of accumulation of material. Exogenous MT reduces the production of ROS (H_2_O_2_ and ${\mathrm{O}}_2^{\cdotp -}$), Na^+^ and Cl^-^, increases K^+^, K^+^/Na^+^ ratio, and proline, and alleviates the oxidative damage, ion toxicity and osmotic stress caused by salt stress to improve the salt tolerance of cotton seedlings. MT increased the compactness of the leaves and the thickness and area of the xylem and phloem of the stems and leaves, promoted the transportation of substances from the underground to the aboveground, and maintained the water and nutrients for the growth of the aboveground parts of the plant. MT can protect the integrity of cotton chloroplast grana lamella structure and mitochondria under salt stress, protect the photosynthetic system of plants, improve Pn, and enhance the salt tolerance of cotton seedlings. The 200 μM MT treatment is the most effective at promoting the growth of cotton seedlings and salt tolerance in this experiment. The result provides a theoretical basis for MT to alleviate salt stress. Further study needs to be conducted to explore the molecular mechanism of the regulation of cotton seedlings under salt stress following treatment with MT.

## Methods

### Plant material and growth conditions

A conventional, widely planted transgenic insect-resistant cotton (*Gossypium hirsutum* L.) cultivar ‘Guoxin No.9’ was used in this study and provided by Guoxin Rural Technical Service Association (Hejian City, China). The cotton seeds were sterilized with 70% ethanol for 30 min, followed by three washes with sterile distilled water to remove the ethanol on surface of the seeds. The sterilized cotton seeds were germinated in the dark in an incubator at 25 °C for 24 h, then sown in plugs of vermiculite and cultivated in a greenhouse (Hebei Agricultural University, Baoding City, Hebei Province, China). The seedlings were cultivated at 28/25 °C (day/night) with a relative humidity of 45 ± 5%, and at a photoperiod (600 μmol m^−2^ s^−1^ light intensity) of 16 h/8 h. At the two-true-leaf stage, the seedlings were transferred to a PVC drum that contained 1.8 L of 1/4 strength complete nutrient solution for hydroponic culture. After 2 days, it was replaced with 1/2 strength complete nutrient solution and incubated for 2 d, then the seedlings were transferred to full-strength nutrient solution for continued cultivation until the end of experiment (Wang et al., 2020). Modified Hoagland solution was the complete nutrient solution used in the experiment for cotton Mengel et al. [84] that consisted of macronutrients (1 mM KH_2_PO_4_, 2 mM MgSO_4_ · 7H_2_O, 4.5 mM NH_4_H_2_PO_4_, and 5 mM KNO_3_), micronutrients (0.02 μM (NH_4_)_6_Mo_7_O_24_ · 4H_2_O, 0.3 μM CuSO_4_ · 5H_2_O, 0.8 μM ZnSO_4_ · 7H_2_O, 5 μM H_3_BO_3_, and 7 μM MnSO_4_ · H_2_O), and other nutrients (0.1 mM EDTA-Na_2_, 0.1 mM FeSO_4_ · 7H_2_O, and 5 mM Ca(NO_3_)_4_ · 4H_2_O). Cotton seedlings were aerated with a small air pump for 1 h each day to ensure sufficient oxygen in the nutrient solution. To keep the ionic components and pH stable in the nutrient solution, the nutrient solutions for each treatment were renewed every 2 d. When the cotton seedlings reached the three-true-leaf stage, the following treatments were imposed:

The seedlings were treated with exogenous MT at various concentrations (50, 100, 200, and 500 μM MT) and subjected to a salt stress treatment. Specifically, the cotton seedlings were sprayed with different concentrations of MT solution until the leaves dripped, once every 24 h for 12 d, respectively. The following experimental groups were arranged into a randomized complete block design with 30 replicates and treated as follows: [1] no MT + no salt treatment (control, CK); [2] no MT + 150 mM NaCl treatment (150 mM NaCl, as determined by the pretest screening) (S); [3] 50 μM MT- sprayed + 150 mM NaCl treatment (S + MT50); [4] 100 μM MT- sprayed + 150 mM NaCl treatment (S + MT100); [5] 200 μM MT- sprayed + 150 mM NaCl treatment (S + MT200); and [6] 500 μM MT- sprayed + 150 mM NaCl treatment (S + MT500). Thirty PVC hydroponic drums were used for each treatment. The physiological indices and microstructure of cotton plants were measured using the third functional leaf (from the top of the plant) at 0, 3, 6, 9, and 12 d after treatment.

### Measurement of growth indicators

The fresh weights of shoots and roots were determined by measurement on an electric balance. The samples were place in an envelope, and then the plants were quenched at 105 °C for 30 min and oven dried at 80 °C for 72 h to a constant weight, which was weighed as the dry weight.

### Measurement of the chlorophyll content, gas exchange parameters, and chlorophyll fluorescence

Chlorophyll content: It was measured with SPAD-502 on the third functional leaf, avoiding the position of cotton veins when measuring. The values were measured at five different positions of cotton leaves, and the average value was calculated.

Photosynthetic characteristics: In sunny weather from 9:00–11:00 and 13:00–15:00, the net photosynthetic rate (Pn), stomal conductance (Gs), transpiration rate (Tr), and intercellular CO_2_ concentration (Ci) of the cotton leaves were measured using a LI-6400 photosynthetic instrument (LI-COR, Lincoln, NE, USA) with a closed-circuit air circuit, built-in light source, CO_2_ concentration of 400 μmol mol^−1^, and light intensity of 600 μmol m^−2^ s^−1^.

Chlorophyll fluorescence parameters: The maximum photochemical efficiency (*Fv/Fm*) of PS II, the actual photochemical quantum yield of PS II (ΦPS II), non-photochemical quenching (NPQ) and apparent electron transfer efficiency (ETR) were measured using a PAM-2500 portable chlorophyll fluorometer (Heinz Walz GmbH, Germany).

### Detection of the Pro content in leaves

The content of proline (Pro) was determined using a Pro assay kit (PRO-1-Y, Suzhou Comin Biotechnology Co., Ltd., Suzhou, China).

### Determination of ion contents

The Na^+^ and K^+^ contents were measured by atomic absorption (ZA3000, Hitachi, Ltd., Tokyo, Japan). The content of Cl^-^ was determined using a Cl^-^ assay kit (Nanjing Jiancheng Bioengineering Institute, Nanjing, China).

### Histochemical detection of H_2_O_2_ and ${\mathrm{O}}_2^{\cdotp -}$ in leaves (histochemical analysis)

H_2_O_2_ and ${\mathrm{O}}_2^{\cdotp -}$ in cotton leaves were detected histochemically by staining with 1 mg/mL DAB (3,3-diaminobenzidine) and 1 mg/mL NBT (nitro-blue tetrazolium), respectively, as described by Chen et al. [85].

### Detection of the contents of H_2_O_2_ and ${\mathrm{O}}_2^{\cdotp -}$ in leaves

The content of hydrogen peroxide (H_2_O_2_) was determined using an H_2_O_2_ assay kit (A064, Nanjing Jiancheng Bioengineering Institute, Nanjing, China). The content of superoxide anions (${\mathrm{O}}_2^{\cdotp -}$) was determined using an ${\mathrm{O}}_2^{\cdotp -}$ assay kit (SA-1-G, Suzhou Comin Biotechnology Co., Ltd., Suzhou, China).

### Fluorescence microscopic analysis of H_2_O_2_ and ${\mathrm{O}}_2^{\cdotp -}$

H_2_O_2_ and ${\mathrm{O}}_2^{\cdotp -}$ were detected with 10 μM 2′,7′-dichlorofluorescin diacetate (DCF-DA) and 10 μM dihydroethidium (DHE), respectively. Both fluorescence probes were prepared by dissolving them in 10 mM Tris−HCl (pH 7.4). After the leaf epidermis was stripped off, they were immediately incubated with the corresponding fluorescence probe at 25 °C for 1 h in the dark. Each sample was quickly washed three times in 10 mM Tris−HCl (pH 7.4) and fixed on a glass slide for observation with a fluorescence microscope (Eclipse Ni-U, Nikon, Tokyo, Japan). Based on these experiments, the 200 μM MT-applied treatment was selected as the most effective. Thus, three treatments could be used in the following experiments: (1) CK (no MT/no salt treatment, control); (2) S (150 mM NaCl) (no MT/150 mM NaCl treatment); (3) S + MT200 (200 μM MT-applied/150 mM NaCl treatment).

### Stomatal structure, density and aperture

A Nikon Microscope (Japan) was used to observe the stoma. The epidermis of the cotton from the third functional leaf was torn off and pasted on a glass slide without a cover glass. Five fields of view for each leaf were randomly selected, and the number of stomata and the morphological characteristics of the stomata were observed under 40× magnification. (1) Stomatal length and width: The stomata for each field of view were measured. The length of the dumbbell-shaped guard cell was the stomata length, and the widest value perpendicular to the dumbbell-shaped guard cell was the stomata width. The average of the length and width of the stomata was calculated to represent the length and width of the stomata in the field of view. (2) Stomatal aperture (SA): All the stomata in each field of view were measured, and the width of the stomata aperture was measured to indicate the stomata opening. The average of the stomatal openings was calculated to represent the stomatal openings in the field of view. (3) Stomata number (SN): The number of stomata on each field of view area was calculated, and the average of the number of stomata in five fields of view represented the number of stomata.

### Transmission electron microscopy (TEM)

The cells were fixed with 2.5% (v/v) glutaraldehyde with phosphate buffer (PB) (0.1 M, pH 7.4) and then washed four times in PB. Then cells were postfixed with 1% (w/v) osmium tetraoxide for 2 h in PB at 4 °C, dehydrated through a graded ethanol series (30, 50, 70, 80, 90, 100, and 100%, 7 min each) into pure acetone (2 × 10 min). The samples were infiltrated in graded mixtures (3:1, 1:1, and 1:3) of acetone and SPI-PON812 resin (Beijing Zhongjingkeyi Technology Co., Ltd., Beijing, China) (16.2 g SPI-PON812, 10 g dodecanoyl succinic anhydride and 8.9 g N-methyl acrylamide), the changed pure resin. Finally, the cells were embedded in pure resin with 1.5% N, N-dimethylbenzylamine and polymerized for 12 h at 45 °C and for 48 h at 60 °C. The ultrathin sections (70 nm thick) were sectioned with a microtome (Leica EM UC6), double-stained with uranyl acetate and lead citrate and examined using transmission electron microscopy (FEI Tecnai Spirit 120kv, FEI Company, Hillsborough, OR, USA).

### Anatomical traits and analyses

To observe the anatomical structure of leaves and stems, they were sampled after 12 days of salt treatment and observed using a paraffin section as described by Mohamed et al. [45]. The third functional leaf and stem were fixed in a solution of formalin-acetic acid-alcohol (FAA) (10 mL formaldehyde + 170 mL 70% (v/v) ethanol + 10 mL glacial acetic acid + 10 mL glycerol) for 72 h under vacuum. The leaf and stem samples were dehydrated and embedded in paraffin wax. A rotary microtome (Zhejiang Jinhua Kedi Instrument Equipment Co., Ltd., Jinhua, China) was used to cut slices of 8 μm thick. The slices were deparaffinized and stained with safranin for 24 h and then washed with ethanol solution to remove excess dye. After decolorizing the sections, they were placed in the solid green dyeing solution for 40 s and then decolorized. A Nikon Microscope was used to observe the microstructural changes of leaves and stems between different treatments.

### Statistical analysis

All the experimental data were analyzed using SPSS 21.0 (IBM, Inc, Armonk, NY, USA) and reported as the mean ± SD (standard deviation) values. The relationships between these traits were analyzed using principle component analysis (PCA), and principle components were obtained on the basis of eigenvalues >1. In order to analyze one-way ANOVA results, a statistical significance threshold of *p* < 0.05 was used in the study.

## Data Availability

The datasets generated and analyzed during the current study are availablefrom the corresponding author on reasonable request.

## Abbreviations

Ci: intercellular CO_2_ concentration
Cor: cortex
DW: dry weight
ETR: electron transport rate
Fv/Fm: maximum potential quantum efficiency of photosystem II
FW: fresh weight
Gs: stomal conductance
H_2_O_2_: hydrogen peroxide
LAP: area of the phloem vessel
LAX: area of the xylem vessel
Lep: lower epidermis
MT: melatonin
NPQ: non-photochemical quenching
$$ {\mathrm{O}}_2^{\cdotp -} $$: superoxide anions
Phv: phloem vessels
Pi: pith
Pp: plastid parenchyma
Pro: proline
ROS: reactive oxygen species
PT: palisade tissue thickness
Pn: net photosynthetic rate
SA: stomatal aperture
SN: stomatal number
SPAD: soil and plant analyzer development
Sp: sponge parenchyma
SPT: stem phloem thickness
ST: spongy tissue thickness
SXT: stem xylem thickness
TEM: transmission electron microscopy
Tr: transpiration rate
Uep: upper epidermis
Xv: xylem vessels
